# Melatonin Secretion Pattern in Critically Ill Patients: A Pilot Descriptive Study

**DOI:** 10.1155/2017/7010854

**Published:** 2017-05-11

**Authors:** Yuliya Boyko, René Holst, Poul Jennum, Helle Oerding, Miki Nikolic, Palle Toft

**Affiliations:** ^1^Department of Anesthesia and Intensive Care Medicine, Odense University Hospital and Southern Danish University, Odense, Denmark; ^2^Institute of Regional Health Service Research, University of Southern Denmark, Odense, Denmark; ^3^Danish Center for Sleep Medicine, Department of Clinical Neurophysiology, Rigshospitalet, and Faculty of Health, University of Copenhagen, Copenhagen, Denmark; ^4^Department of Anesthesia and Intensive Care Medicine, Vejle Hospital, Vejle, Denmark; ^5^Faculty of Health, Southern Danish University, Odense, Denmark; ^6^Danish Center for Sleep Medicine, Department of Clinical Neurophysiology, Rigshospitalet, Copenhagen, Denmark

## Abstract

Critically ill patients have abnormal circadian and sleep homeostasis. This may be associated with higher morbidity and mortality. The aims of this pilot study were (1) to describe melatonin secretion in conscious critically ill mechanically ventilated patients and (2) to describe whether melatonin secretion and sleep patterns differed in these patients with and without remifentanil infusion. Eight patients were included. Blood-melatonin was taken every 4th hour, and polysomnography was carried out continually during a 48-hour period. American Academy of Sleep Medicine criteria were used for sleep scoring if sleep patterns were identified; otherwise, Watson's classification was applied. As remifentanil was periodically administered during the study, its effect on melatonin and sleep was assessed. Melatonin secretion in these patients followed a phase-delayed diurnal curve. We did not observe any effect of remifentanil on melatonin secretion. We found that the risk of atypical sleep compared to normal sleep was significantly lower (*p* < 0.001) under remifentanil infusion. Rapid Eye Movement (REM) sleep was only observed during the nonsedation period. We found preserved diurnal pattern of melatonin secretion in these patients. Remifentanil did not affect melatonin secretion but was associated with lower risk of atypical sleep pattern. REM sleep was only registered during the period of nonsedation.

## 1. Introduction

Disturbances in sleep and circadian rhythm in critically ill patients have been in focus during recent years due to potential association of disrupted sleep with delirium and increased morbidity and mortality in this patient group.

Critically ill patients are exposed to a range of factors that may affect the brain structures responsible for the maintenance of circadian and sleep homeostasis: the brain stem, hypothalamus, and thalamus. Morbidities and medication, for instance, sedative agents, are among the important factors.

Melatonin is the hormone secreted by the pineal gland, located in epithalamus, under control of the suprachiasmatic nucleus. Melatonin takes part in the regulation of sleep and circadian rhythm. Detection of melatonin levels in serum or saliva or melatonin-derivate 6-sulfatoxymelatonin (6-SMT) in urine is used to estimate melatonin secretion pattern.

Melatonin secretion in critically ill patients has been investigated in several studies reporting conflicting results. Some studies reported abolished melatonin secretion pattern in sedated critically ill patients [1–3], while preserved circadian periodicity of melatonin secretion in critically ill patients with [4] or without [2] sedation was reported in other studies.

The gold standard for sleep monitoring, allowing for the description of sleep patterns, is polysomnography.

Previous polysomnographic sleep studies in intensive care unit (ICU) have shown severely abnormal sleep patterns. Opioid agents (e.g., fentanyl), which are often used for sedation in critically ill patients, have been shown to reduce restorative sleep [5]. However, no polysomnographic studies have been done to show the effect of remifentanil, the ultrashort acting synthetic opioid, used for analgosedation, on sleep pattern in critically ill patients.

In this pilot descriptive study we aimed (1) to describe melatonin secretion pattern in conscious critically ill patients on mechanical ventilation and (2) to describe whether melatonin secretion and sleep patterns as determined by PSG differed in these patients with and without remifentanil infusion. Remifentanil is a standard drug of choice for analgosedation in the ICU, where the study was carried out.

## 2. Materials and Methods

This pilot study was conducted in a mixed 8-bed Intensive Care Unit at Vejle Hospital, Denmark. It was approved by the Regional Ethics Committee of Southern Denmark (S-20120001). The patients included in the study were a part of the “Sleep in ICU” project, registered with ClinicalTrials.gov (NCT01681043).

Conscious critically ill adults with expected duration of mechanical ventilation ≥ 48 hours were eligible for the study. The exclusion criteria were coma, delirium at inclusion, acute clinical signs of intracerebral events, and circulatory shock. Eligible patients were informed about the project by the main investigator and included in the study after informed consent.

The patients had an arterial catheter routinely inserted at admission to the ICU. Blood samples from this catheter were taken in 8 patients every 4th hour in a 48-hour period to assess S-melatonin. Enzyme-linked immunosorbent assay (ELISA) method was used for melatonin analysis, using ELISA kit provided by IBL international [6].

PSG monitoring included recording of electroencephalography (EEG), bilateral electrooculography, and submental electromyography according to the worldwide accepted standard of American Academy of Sleep Medicine (AASM) for polysomnographic sleep measurement. The EEG consisted of frontal (F3-A2, F4-A1), central (C3-A2, C4-A1), and occipital (O1-A2, O2-A1) channels and was recorded using the EEG and Sleep Recorder Trackit (Lifelines Ltd., UK). The electrodes were applied by the main investigator during the first day of participation. All PSGs consisted of 30 sec epochs and were manually assessed by an expert in sleep medicine. Standard AASM sleep assessment criteria could not be used alone due to abnormal patterns in many of the recordings. Accordingly, the modified sleep scoring classification for critically ill patients suggested by Watson et al. was applied [7]. Behavioral aspect of Watson's classification was not used because the recordings were reviewed later.

## 3. Statistical Analysis

Log transformation was used for normalizing the data. A random effects linear regression model was used to assess the effects of remifentanil (as a categorical variable), age, sex, and Sequential Organ Failure Assessment (SOFA) score [8] upon the melatonin secretion. Trigonometric terms were included to account for the diurnal harmonic variation. Inhomogeneity between patients was accounted for by using patients' id as a random effect. Insignificant effects were removed one at a time, followed by a reestimation. Effects with *p* values < 0.05 were considered significant. Graphs of the observed data, plotted against time, suggested that the level of melatonin had a diurnal variation in the form of a harmonic curve. By fundamental trigonometric lows, such a curve can be achieved by a sum of a sine and a cosine term: *a∗*sin⁡(*x*) + *b∗*cos⁡(*x*), where *a* and *b* determine the time of nadir/acrophase as well as the amplitude of the curve. We entered gender-specific sin⁡(time) and cos⁡(time) terms in the regression. The estimated coefficients were subsequently used to calculate gender-specific estimates of time for nadir/acrophase and amplitude.

The recorded PSG epochs were classified into four groups: normal sleep (scored according to AASM standard), atypical sleep (Watson's classification), awake, and undefined (the epochs with artefacts). A multinomial regression model with random effects, including remifentanil (as a categorical variable), sex, and age as covariates, was used for testing the association between remifentanil infusion and the four sleep/wake conditions.

Normal sleep and atypical sleep groups were plotted as the proportions of sleep (normal sleep + atypical sleep + wake + undefined = 100%) over the 24-hour period together with melatonin to visualize diurnal variation of sleep and melatonin. Melatonin line represents connected mean melatonin values at each sample point (six sample points with 4 hour intervals), and these values were standardized to sleep proportion values to visualize both curves on the same scale on *y*-axis so that melatonin_std_ = (melatonin/max(melatonin)) × max(sleep, %).

STATA, version 13 and 14, was used as statistical software [9].

## 4. Results

Eight conscious, critically ill patients on mechanical ventilation and being able to give consent were included in the study. Data collection proceeded from September 2012 until November 2013. Patient baseline characteristics are presented in Table 1.

Remifentanil infusion was applied as analgosedation in 6 patients, dose of 0.06 to 0.360 mg/hour, either during the whole period of participation or episodically after decision of an intensivist doctor in charge. Remifentanil was infused during 43% of the total time of participation, while no continuous sedation was given during the rest (57%) of the time.

**Figure 1:**
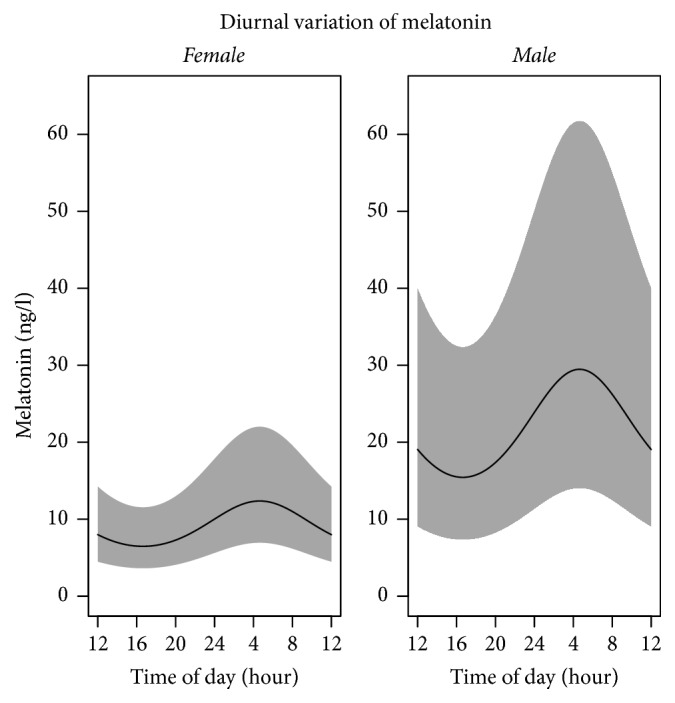
Diurnal variation of melatonin by gender. Mean melatonin values, ng/L; the grey field represents confidence intervals.

**Figure 2:**
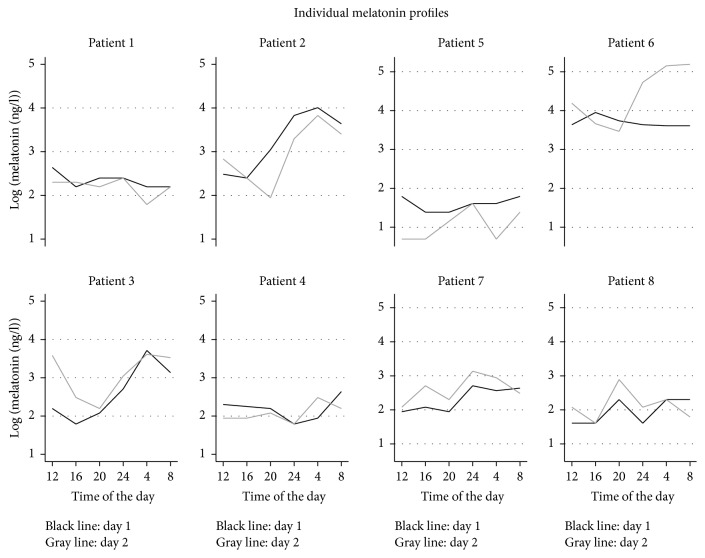
Individual melatonin profiles (8 patients, 2 days for each patient). The log-scale of melatonin is used to allow for appreciation of both interindividual and diurnal variation patterns.

The analysis of melatonin secretion showed that critically ill patients followed a diurnal harmonic curve, phase-delayed compared with the physiological norm pattern of melatonin secretion [10] with acrophase at approximately 4:30 am and nadir at approximately 4:30 pm (Figure 1). The interindividual variation and the interday variation within individuals contributed with 64% and 10%, respectively, of the total random variation.  Figure 2 gives the diurnal variation of melatonin by patients for both days (individual melatonin profiles). The log-scale was found to normalize the data, and it is on this scale that data allow for appreciation of both interindividual and diurnal variation patterns. On the original scale the dominant interindividual variation eliminates the diurnal variation for patients at low levels of melatonin. A borderline significant effect of sex (*p* = 0.06) was found on melatonin secretion pattern. Remifentanil infusion was not significantly associated with the melatonin secretion pattern, whereas the diurnal variation was significant.

**Figure 3:**
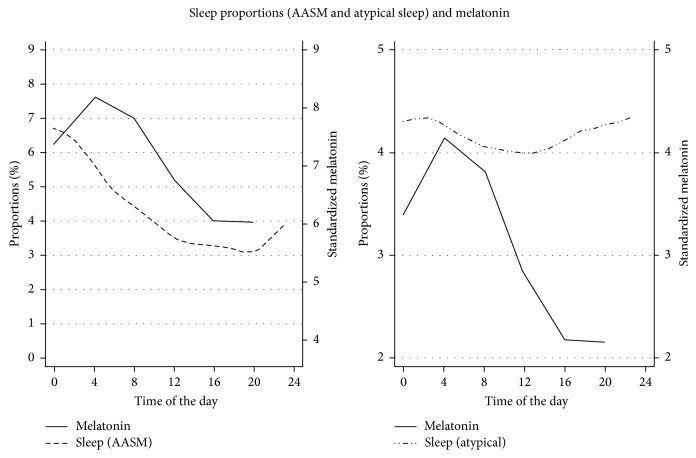
Diurnal variation of sleep and melatonin. Melatonin line represents connected mean melatonin values at each sample point. These values are standardized to sleep proportion values to visualize both curves on the same scale on *y*-axis. Normal sleep (AASM standard) and atypical sleep (Watson's classification) are plotted as the proportions of sleep (normal sleep + atypical sleep + wake + undefined = 100%) over the 24-hour period together with melatonin to visualize diurnal variation of sleep and melatonin.

Diurnal variation of melatonin and sleep (% normal sleep, scored according to AASM standard, and % atypical sleep, scored according to Watson's classification) are presented in Figure 3.

**Figure 4:**
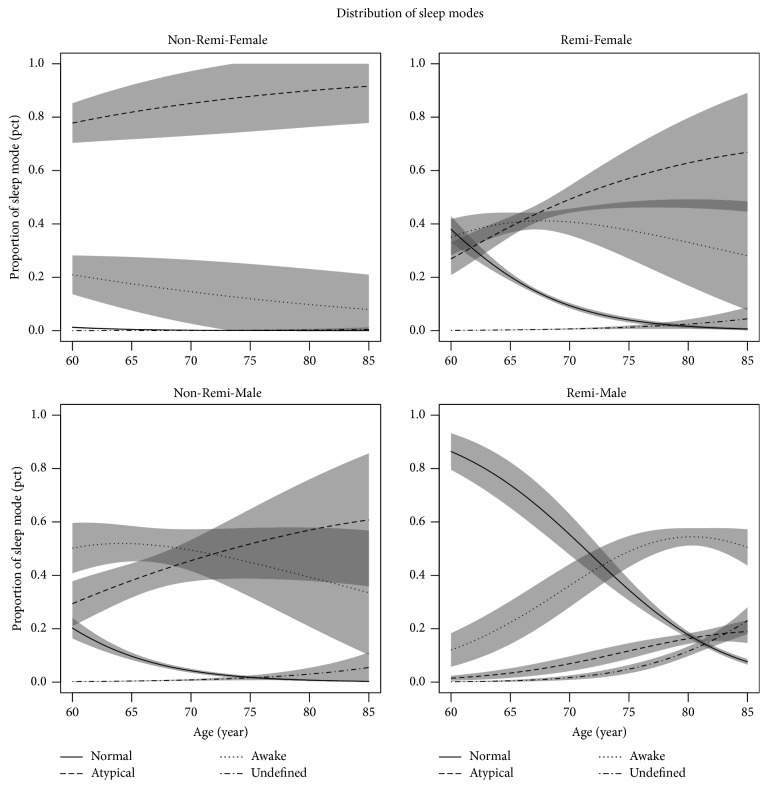
Distribution of sleep modes by gender and remifentanil infusion. All the PSG epochs are classified into 4 groups: normal sleep (AASM standard), atypical sleep (Watson's classification), awake (wake), and undefined (the epochs with artefacts). The distribution of these groups by gender and remifentanil infusion at different ages is shown on the graph. The grey field represents confidence intervals.

The analysis of association of remifentanil analgosedation and sleep patterns showed that remifentanil infusion affected the balance between the four groups (normal sleep, atypical sleep, wake, and undefined) significantly but in different manners for males and females (Figure 4). Furthermore, there was a strong age effect, which also varied between genders and between remifentanil infusion and noninfusion.

Use of remifentanil infusion was associated with the reduced proportion of atypical sleep for females at all ages, with a slight increase at increasing ages. Females of age < 70 years increased their proportion of normal sleep and females at all ages increased their proportion at wake condition.

Remifentanil infusion was associated with a significant reduction of atypical sleep in males as well, but at a lower level compared with females. The proportion of time being awake was reduced for males < 70 years but increasing with age to higher level relative to not being under remifentanil infusion. The most notable effect was a large increase in the proportion of normal sleep, in particular for males < 70 years, starting at 85% at age 68 and declining to 10% for males aged 85.

REM sleep was only observed in the PSG recordings during the period without remifentanil in two patients (4.5 min and 10.5 min, resp.).

Atypical stages 1 and 2, scored in accordance with Watson's modified sleep scoring classification for critically ill patients [7] (presence of alfa or theta activity and delta activity), were registered with or without remifentanil infusion, while atypical stages 3–5 (polymorphic delta activity without alfa or beta activity, burst-suppression pattern, or suppressed EEG pattern) were only observed during remifentanil infusion.

## 5. Discussion

The main findings of this pilot descriptive study were as follows:

- melatonin secretion pattern in conscious, critically ill mechanically ventilated patients followed a harmonic sinusoid diurnal but phase-delayed curve;
- no association of remifentanil infusion with melatonin secretion pattern was observed;
- remifentanil infusion was associated with lower risk of atypical sleep compared with normal sleep.

Melatonin secretion pattern has been investigated in several studies, reporting controversial findings.

Disturbed diurnal variation of melatonin secretion in critically ill patients was found in several studies [1–3, 11–13]. Shilo et al. described missing nocturnal top of melatonin in conscious ICU patients [11]. Frisk et al. found association of lower 6-SMT (6-sulfatoxymelatonin, the major melatonin metabolite, measured in urine) excretion with mechanical ventilation and higher 6-SMT levels associated with benzodiazepine sedation, but not with propofol or opioids [12]. Abolished melatonin secretion pattern in sedated mechanically ventilated patients was shown in the study by Olofsson et al., but no correlation between levels of sedation and melatonin levels was found though [3]. Benzodiazepines, opioids and propofol were used for sedation. Disturbed diurnal pattern of melatonin secretion was found in sedated critically ill patients with and without brain injury in the study by Paul and Lemmer, where serum melatonin levels were lower in the brain injury group [1]. Benzodiazepines and opioids were used for sedation. Disturbed circadian rhythmicity of melatonin secretion was shown in septic patients in two studies [2, 13]. The majority of patients (5 out of 7) in the study by Verceles et al. were on mechanical ventilation [13]. Two patients received propofol sedation and one patient was sedated with dexmedetomidine. All the patients in the sepsis group in the study by Mundigler et al. were on mechanical ventilation and sedated with benzodiazepine and opioid [2]. However, preserved diurnal pattern of melatonin secretion was registered in the group of patients without sepsis in this study.

Inconsistently with the above-mentioned studies, preserved circadian rhythmicity of melatonin secretion in critically ill patients was described in two studies [4, 14]. Riutta et al. found maintained diurnal pattern of melatonin secretion in nonseptic critically ill patients, where 20 out of 40 patients were on mechanical ventilation and 25 patients were sedated with benzodiazepines [14]. Benzodiazepines did not affect diurnal variation of 6-SMT excretion. Collection of twelve-hour urine samples allowed for assessment of diurnal rhythm but not the precise timing for 6-SMT excretion. In difference, Gehlbach et al. measured 6-SMT levels in urine, collected with hourly intervals, and found preserved circadian rhythm in the majority of sedated critically ill patients on mechanical ventilation (13 out of 16), but only 4 patients had normal timing [4]. Opioids, propofol, and benzodiazepines were used for sedation. Most subjects exhibited phase-delayed excretion. There was no difference between patients with and without sepsis in the proportion of patients with normal circadian timing.

In line with findings by Gehlbach et al. [4], we observed phase-delayed diurnal variation of melatonin secretion in our patients, with acrophase in serum at approximately 4.30 am and nadir at 4.30 pm. Evening light exposure in ICU due to procedures could probably explain phase-delay in our patients. Abolished regulation of melatonin secretion by light and darkness in critically ill patients of different age groups was described in the study by Perras et al. [15], and the similar finding is reported in the study by Verceles et al. [13] in patients with severe sepsis, where light levels were shown not to entrain circadian rhythm. Critically ill patients with different levels of consciousness were used in these studies, while all the patients in our study were conscious and able to open their eyes, and no eyeshades were used. Unfortunately, we did not register light levels in our study.

In alignment with previous knowledge [16], we found considerable interindividual variation (64%) and a slight effect of sex on melatonin secretion pattern. No age effect was seen though, probably explained by a small sample size.

The absence of correlation between severities of critical illness scores with melatonin secretion was mentioned in some studies [4, 12, 14]. Perras et al. reported APACHE scores to be negatively correlated with melatonin concentrations in septic patients but found no correlation in patients without sepsis [17]. We did not observe any effects of SOFA scores on melatonin secretion pattern.

The role of sedation related to melatonin secretion is not completely understood. Different sedative agents were used in the above-mentioned studies in critically ill patients showing no clear association between the level of sedation or the type of sedative agent and melatonin secretion pattern. Some of the patients in our study were analgosedated with small doses of remifentanil during the whole or a part of the study period. We found no significant effect of remifentanil on melatonin secretion pattern. To our knowledge, this is the first time melatonin secretion pattern is investigated under remifentanil infusion in critically ill patients. Nevertheless, our results are in line with the study by Bonafide et al., where melatonin secretion was investigated in healthy participants under sedation with remifentanil and found preserved [18]. The relatively small doses of remifentanil used in both studies may possibly explain the missing effect of remifentanil on melatonin secretion.

Graphical exploration of circadian rhythmicity and sleep in these critically ill patients suggests the presence of diurnal variation in sleep, phase-delayed compared with the physiological norm, in the patients with normal sleep pattern. No rhythmicity or association with melatonin was seen in the patients with atypical sleep (Figure 3). Due to the small sample, current data do not allow for a more rigorous analysis. The potential association between sleep pattern and the diurnal production of melatonin ought to be studied in a larger sample of critically ill patients.

In animal studies, opioids have been described to disrupt sleep architecture and reduce restorative sleep by affecting different sleep regulating centers in the brain. Morphine and fentanyl were found to decrease acetylcholine in pons, the region regulating REM sleep [5]. Morphine was shown to affect mu and kappa receptors in ventrolateral preoptic area, the region primarily responsible for non-REM sleep [19]. However, remifentanil was not found to have the same effect on acetylcholine as morphine and fentanyl in the study by Mortazavi et al. [5]. Bonafide et al. investigated the effect of remifentanil on sleep in healthy humans and found reduced REM sleep [18]. In line with this study, we only observed REM sleep in the epochs recorded under the period without remifentanil.

To our knowledge, the association of remifentanil infusion with the presence of atypical/normal sleep characteristics in PSG in critically ill patients has not yet been studied. Surprisingly, we found remifentanil to be associated with lower risk for atypical sleep patterns compared with normal sleep. The etiology of this finding is unclear and needs further investigation.

The main weakness of this study is the small number of patients, since inhomogeneity between patients constitutes the major source of random variation. This is to some extent alleviated by multiple measurements on each patient and thereby allows for detection of diurnal variation of melatonin secretion.

Melatonin was measured in serum, which is the best way to estimate circadian function. However, the analysis of circadian rhythm could be more accurate, if the sampling intervals were shorter.

We did not measure light levels in the patient rooms for technical reasons, which is another limitation of this study.

We found remifentanil infusion to be significantly associated with an increased proportion of normal sleep. However, this study was observational and relatively small doses of remifentanil were used.

As the sample size was small (8 patients), we did not account for the admission diagnoses in our statistical analysis. Despite the variety of diagnoses at admission, all the patients were conscious, critically ill mechanically ventilated adults without circulatory failure. Accordingly, we found this sample to be potentially representative for a specific group of critically ill patients.

Despite the many limitations, this study reveals some interesting findings and should therefore be considered hypothesis-generating. A larger randomized trial to clarify the influence of remifentanil analgosedation on melatonin and sleep patterns in critically ill patients is recommended. The impact of the remifentanil dose ought to be studied as well. We suggest the registration of light levels as well as hourly melatonin sampling to be included in the method in future studies to improve the understanding of the phase-shift of circadian rhythm. The addition of video recording to PSG would be useful for the complete assessment of sleep/wake states in critically ill patients and would allow for the evaluation of behavioral aspects.

*Conclusions.* In this pilot descriptive study, melatonin secretion pattern in conscious, critically ill patients on mechanical ventilation was found to follow a diurnal but phase-delayed curve. Remifentanil infusion did not affect melatonin secretion but was associated with lower risk of atypical sleep. The mechanism of this finding is unknown. REM sleep was only observed in the PSG recordings without remifentanil.

## Figures and Tables

**Table 1:** Patient baseline characteristics, *n* = 8.

Age, years, median (min; max)	70 (62; 85)
Sex	Female: 5, Male: 3
APACHE II^1^, median (min; max)	24 (21; 33)
SOFA^2^, median (min; max)	3 (2; 5)
RASS score^3^ (range)	−1 to +1
Length of ICU stay before the inclusion, days, median (min; max)	2.5 (1; 10)
Admission diagnosis (%)	
Pneumonia	3 (40%)
Chronic obstructive pulmonary disease	3 (40%)
Sepsis/respiratory distress	1 (10%)
Neurologic disease	1 (10%)

^1^APACHE II: Acute Physiology and Chronic Health Evaluation II (severity of disease classification system).

^2^SOFA score: Sequential Organ Failure Assessment score.

^3^RASS-score: Richmond Agitation-Sedation Scale score.
